# Objectively measured preoperative physical activity and sedentary behaviour among Finnish patients scheduled for elective cardiac procedures: baseline results from randomized controlled trial

**DOI:** 10.1186/s13102-022-00522-1

**Published:** 2022-07-16

**Authors:** Sini Vasankari, Juha Hartikainen, Ville Vasankari, Vesa Anttila, Kari Tokola, Henri Vähä-Ypyä, Pauliina Husu, Harri Sievänen, Tommi Vasankari, Jari Halonen

**Affiliations:** 1 Derpartment of Clinical Medicine, University of Turku, Turku, Finland; 2 Heart Center, Kuopio University Hospital (KUH), Kuopio, Finland; 3 Department of Neurosurgery, Helsinki University Hospital, University of Helsinki, Helsinki, Finland; 4 Heart Center, Turku University Hospital, Turku, Finland; 5 The UKK Institute for Health Promotion Research, Tampere, Finland; 6 The Faculty of Medicine and Health Technology, Tampere University, Tampere, Finland

**Keywords:** Accelerometry, Aortic valve stenosis, Coronary artery disease, Mitral valve insufficiency, Physical activity, Sedentary behavior

## Abstract

**Background:**

We investigated preoperative physical activity (PA) and sedentary behaviour (SB) in patients scheduled for elective cardiac procedures and compared them with population-based sample of Finnish adults.

**Methods:**

Cardiac patients (n = 139) undergoing cardiac operations carried a triaxial accelerometer for seven days during the month before the procedure. Patients were categorised into four groups according to the procedure: percutaneous coronary intervention or coronary angiography (PCI-CA), coronary artery bypass grafting (CABG), aortic valve replacement (AVR) and mitral valve surgery (MVS). The raw accelerometer data was analyzed with dedicated algorithms to determine metabolic equivalents (METs, 3.5 mL/kg/min of oxygen consumption) of PA. The intensity of PA was divided into two categories: light (LPA, 1.5–2.9 METs) and moderate-to-vigorous (MVPA, ≥ 3.0 METs), while SB represented intensity < 1.5 MET without movements. SB and PA were described as daily means and accumulation from different bout lengths. Daily standing, steps and mean and peak MET-values were calculated. The results were compared between the patient groups and against the reference group from a population-based study FinFit2017.

**Results:**

Cardiac patients had fewer daily steps than the FinFit population (*p* = 0.01), and less SB accumulating from < 20 min bouts (*p* = 0.002) but more from 20 to 60 min bouts (*p* = 0.002). Particularly, CABG group had less daily MVPA (*p* = 0.002) and MVPA accumulating from > 10 min bouts (*p* < 0.001) than the FinFit population.

**Conclusions:**

We found large differences in PA and SB between the patient groups and the FitFit population, CABG group having the worst activity profile. Also, the variation within the patient groups was wide, which should be considered to individualise the rehabilitation programs postoperatively.

*Trial registration* clinicaltrials.gov (NCT03470246). Registered 19 March 2018, https://clinicaltrials.gov/ct2/show/NCT03470246.

## Background

Cardiovascular diseases (CVDs) account for almost one-third of deaths globally being the number one cause of death [1]. Coronary artery disease is the most common CVD [2, 3]. Aortic valve stenosis and mitral valve insufficiency are CVDs with increasing prevalence and limited possibilities for conservative treatment [2, 4]. Besides lifestyle and medical therapy, invasive procedures, such as percutaneous coronary intervention (PCI), coronary artery bypass grafting (CABG), aortic valve replacement (AVR), mitral valve replacement (MVR) and mitral valve repair (MVP) are sometimes necessary options for these patients [5]. However, these operations also cause substantial costs to health care system, and non-invasive, pre- and postoperative interventions could be applied as adjuvant tools to optimize the treatment and rehabilitation of these patients [5].

Physical activity (PA), defined as energy expenditure > 1.5 metabolic equivalents (METs) related to body movement, has been recognized as an important contributor to both prevention and treatment of CVDs [6–9]. Correspondingly, physical inactivity (not meeting the PA guidelines) has been reported to be a risk factor for CVDs [10]. In addition, low level of PA is associated with an increased risk of immediate postoperative complications after cardiac surgery [11]. Poor cardiorespiratory fitness is also an independent risk factor for CVDs [12, 13] whereas good preoperative cardiorespiratory fitness predicts higher survival after cardiac surgery [14].

Sedentary behaviour (SB) has also been found as a risk factor for CVDs [15, 16]. The definition of SB is energy expenditure ≤ 1.5 METs in lying or sitting position [17]. Higher overall sedentary time and the number of SB bouts have been reported to associate with increased CVD risk [15, 16]. There is little data on the impact of SB in CVD secondary prevention, such as rehabilitation after cardiac operations [18]. Both preoperative PA and SB may separately have direct associations with mortality after cardiac surgery [11, 19]. However, the amount of objective data on these is insufficient.

Traditionally, estimation of PA and SB have been based on questionnaires. However, they have been shown to have limited reliability and validity [20]. Therefore, device-based methods have become the state-of-the-art in activity monitoring [20]. For instance, analyzing raw accelerometer data with algorithms such as the mean amplitude deviation (MAD) and the angle for posture estimation (APE) can be used to estimate PA and SB with high accuracy and comparability [21–23]. In this study, we used these algorithms to objectively investigate preoperative PA and SB among patients scheduled for elective PCI or coronary angiography (PCI-CA), CABG, AVR or mitral valve surgery (MVS). In addition, we compared their PA and SB to that of general Finnish population.

## Methods

### Participants

**Fig. 1:**
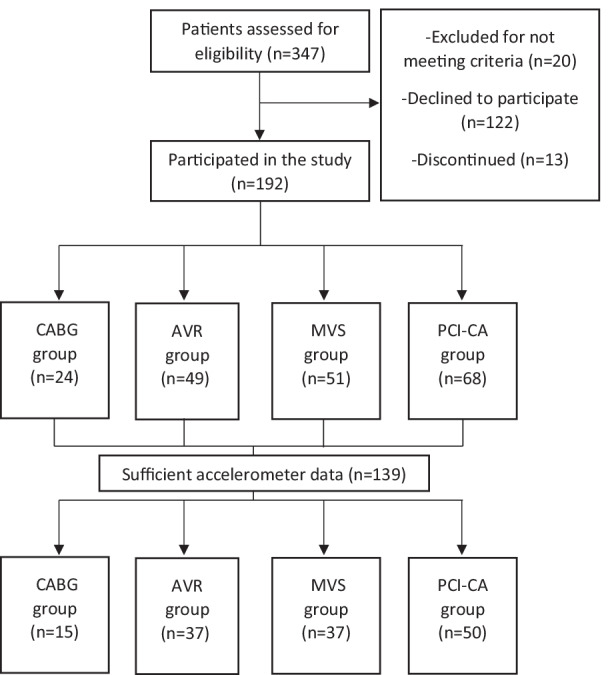
Study participation flow diagram. CABG: coronary artery bypass grafting; AVR: aortic valve replacement; MVS: mitral valve surgery; PCI-CA: percutaneous coronary intervention or coronary angiography

This study is based on the baseline measurements of the ”Personalized intervention to increase physical Activity and reduce sedentary behaviour in rehabilitation after Cardiac Operations (PACO)” trial [24]. The data was collected between May 2018 and November 2020. Patients scheduled for elective PCI-CA, CABG, AVR or MVS (MVP or MVR) were asked to participate in the trial. The patients carried an accelerometer 24/7 during seven consecutive days. Four groups were formed according to the performed operation: PCI-CA, CABG, AVR and MVS. The patients, who were scheduled for combined CABG and valve surgery (CABG + AVR or CABG + MVS), were included in the valvular surgery group in question (AVR or MVS, respectively) [19], because valvular surgery was considered more invasive than CABG. The criterion for sufficient using of the accelerometer was 24 h for at least four days (Fig. 1).

### Comparison with population-based sample

A population-based sample of 60-69-year Finnish adults from the FinFit2017 study, was employed as the reference group for the cardiac procedure patients [25]. The FinFit2017 study was chosen as the reference, because the same 24/7 accelerometer measurements and analysis methods were used, it represents the general population of Finnish adults, and the data collection period was during 2017–2019, which is close to that of the cardiac patients. The age group from 60 to 69 years was selected, as the majority of patients in all cardiac patient groups in this study were within that age range.

### Measurement of physical activity and sedentary behaviour

Patients’ PA and SB were recorded using a triaxial accelerometer (UKK RM42, UKK Terveyspalvelut Oy, Tampere, Finland) for seven consecutive days during the month preceding the scheduled cardiac procedure. During waking hours, the accelerometer was attached to an elastic belt and worn on the right side of the hip, except in shower and other water activities. When going to bed for sleep, the accelerometer was moved from the belt to an adjustable wrist band and attached to the nondominant wrist [24]. Participants received both oral and written instructions on using the accelerometer and changing the attachment point. The raw data was transferred from the accelerometers to a hard drive for further analysis.

The MAD values were calculated in 6-second epochs from the resultant acceleration (the vector sum of the three orthogonal acceleration components). The MAD algorithm is an accurate predictor of VO_2_ and energy consumption during locomotion [21, 22], and thus, allows conversion from MAD values into METs (MET, 3.5 mL/kg/min of oxygen consumption). One-minute exponential moving average of MET values from 6 s epochs was determined, and further used to estimate the intensity of PA.

PA was categorized into three intensity levels: light (1.5–2.9 METs), moderate (3.0-5.9 METs) and vigorous (> 6.0 METs) [6]. Moreover, these were combined into two types of PA: light (LPA, 1.5–2.9 METs) and moderate-to-vigorous (MVPA, ≥ 3.0 METs) [16].

SB was defined as energy expenditure ≤ 1.5 METs while sitting or in reclined position and standing as energy expenditure ≤ 1.5 METs in the upright position [17]. These three different body postures were recognized accurately with the APE analysis [23]. The APE was based on the comparison of the incident accelerometer orientation with the reference vector of upright position, which was determined in relation to the Earth’s gravity vector while walking [23].

The number of daily steps was calculated from the accelerometer data [23]. Using the methods described earlier, the number and accumulated time of separate bout lengths of PA, SB and standing were determined, besides their overall time [16]. In addition, the peak and mean daily 3-min MET levels were estimated [16].

### Statistical analysis

Characteristics of the patients are shown as means with standard deviations for numerical variables and counts with percentages for categorical variables. Kruskall–Wallis test and Fisher’s Exact test were used to test differences between treatment groups for characteristics. Treatment group differences for the accelerometer variables were tested with Analysis of Covariance using Sidak-adjustment to correct for multiple comparisons. Differences between PACO treatment groups and FinFit2017 were tested with independent samples t-test assuming that variances are not equal. Fisher’s Exact tests were conducted in R (R Core Team, 2020) and other analyses in SPSS 27 (IBM Corp. 2020, Armonk, NY).

## Results

**Fig. 2:**
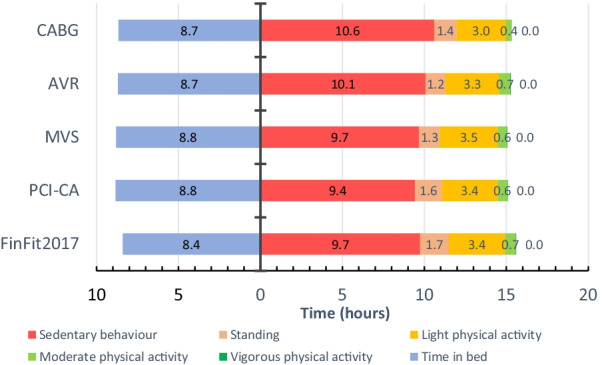
Average time spent sleeping, and during wake time in physical activity and sedentary behaviour in different patient groups and FinFit2017 population sample. CABG: coronary artery bypass grafting; AVR: aortic valve replacement; MVS: mitral valve surgery; PCI-CA: percutaneous coronary intervention or coronary angiography; FinFit2017: population-based sample of 60-69-year-old Finnish adults.

A total of 347 patients scheduled for elective PCI-CA, CABG, AVR or MVS were asked to participate in the trial, of which 192 patients participated. The group sizes were: (1) PCI-CA (n = 68), (2) CABG (n = 24), (3) AVR (n = 49) and (4) MVS (n = 51). Of those, 139 (PCI-CA: 50; CABG: 15; AVR: 37; MVS: 37) met the criterion for sufficient using of the accelerometer. Characteristics, clinical variables and medications of the patients are presented in Table 1. For example, diabetes, hypertension and hypercholesterolemia were variables that had significant differences between patient groups. The variation in accelerometer measurement variables within the patient groups is depicted in Table 2. The mean daily accumulated time of the four patient groups was 22-40 min in moderate PA, 0.0-1.6 min in vigorous PA and 9 h 26 min-10 h 36 min in SB (Fig. 2; Table 2). Among the AVR group patients, the longest mean daily MVPA time was 18 times as much as the shortest one. In all patient groups, the patient with most steps per day had at least six times as many steps as the patient with the smallest daily number. In the MVS group, the patient with the greatest average daily three-minute mean MET-level, had threefold MET-level compared to the smallest one (Table 2).

**Table 1:** Characteristics of the patients

	CABG (n = 8–24)	AVR (n = 22–49)	MVS (n = 22–51)	PCI-CA (n = 28–68)	p-value
Age (y)	65.4 (6.5)	63.0 (11.4)	60.4 (11.3)	66.0 (6.2)	0.056
Male*	17 (70.8)	35 (71.4)	44 (86.3)	47 (69.1)	0.14
BMI (kg/m^2^)	30.0 (4.7)	28.5 (5.2)	26.6 (4.6)	27.5 (4.0)	0.021
Systolic blood pressure (mmHg)	136.0 (13.5)	137.3 (17.3)	138.8 (12.8)	141.0 (16.6)	0.69
Diastolic blood pressure (mmHg)	79.2 (11.6)	73.6 (13.9)	81.3 (9.1)	88.9 (74.2)	0.077
Total cholesterol (mmol/l)	3.7 (0.9)	4.0 (0.9)	4.4 (1.0)	3.9 (1.1)	0.048
HDL cholesterol (mmol/l)	1.33 (0.27)	1.50 (0.32)	1.47 (0.32)	1.39 (0.45)	0.16
LDL cholesterol (mmol/l)	2.12 (0.86)	2.40 (0.77)	2.80 (1.03)	2.21 (0.96)	0.032
Triglycerides (mmol/l)	1.05 (0.39)	1.01 (0.40)	1.21 (0.50)	1.41 (0.87)	0.13
Smoking*	1 (4.2)	1 (2.2)	1 (2.0)	3 (4.5)	0.80
Diabetes*	15 (62.5)	10 (21.3)	2 (3.9)	18 (26.9)	< 0.001
Hypertension*	22 (91.7)	27 (57.4)	19 (38.0)	45 (68.2)	< 0.001
Hypercholesterolemia*	21 (87.5)	30 (66.7)	34 (68.0)	63 (94.0)	0.002
Atrial fibrillation*	3 (13.6)	9 (20.0)	10 (19.6)	8 (12.3)	0.59
Heart failure*	0	2 (4.3)	2 (3.9)	1 (1.5)	0.69
Coronary artery disease*	24 (100)	14 (29.8)	15 (29.4)	52 (77.6)	< 0.001
Arteriosclerosis obliterans*	2 (8.3)	0	0	0	0.016
Stroke or transient ischemic attack*	2 (8.3)	4 (8.5)	3 (5.9)	4 (6.1)	0.89
Myocardial infarction*	6 (25.0)	0	0	10 (15.4)	< 0.001
Previous percutaneous coronary intervention*	9 (37.5)	4 (8.5)	3 (5.9)	21 (31.8)	< 0.001
Previous CABG*	0	0	0	3 (4.5)	0.17
Previous valve surgery*	0	2 (4.3)	2 (3.9)	1 (1.5)	0.74
Pacemaker*	1 (4.2)	2 (4.3)	1 (2.0)	1 (1.5)	0.64
Lung disease*	6 (25.0)	8 (17.4)	7 (13.7)	10 (15.2)	0.63
Cancer*	0	0	1 (2.0)	6 (9.1)	0.058
Thyroid gland disease*	2 (8.3)	2 (4.3)	1 (2.0)	8 (11.9)	0.15
LVEF (%)	59.5 (9.1)	57.4 (10.4)	65.7 (10.7)	60.6 (8.6)	0.001
Medication*					
Beta blocker	18 (75.0)	17 (36.2)	16 (31.4)	44 (65.7)	< 0.001
Calsium blocker	10 (41.7)	12 (26.1)	9 (17.6)	15 (22.4)	0.16
ACE inhibitor/ Angiotensin receptor blocker	17 (70.8)	26 (55.3)	20 (39.2)	36 (53.7)	0.070
Acetylsalicylic acid	18 (75.0)	20 (42.6)	15 (29.4)	47 (70.1)	< 0.001
Adenosine-diphosphate receptor antagonists	4 (16.7)	3 (6.4)	2 (3.9)	3 (4.5)	0.16
Warfarin	1 (4.2)	3 (6.5)	4 (7.8)	5 (7.5)	1.0
Novel oral anticoagulant	3 (12.5)	3 (6.4)	6 (11.8)	5 (7.6)	0.66
Statin	21 (87.5)	30 (63.8)	28 (54.9)	55 (82.1)	0.002
Ezetimibe	7 (29.2)	3 (6.4)	4 (7.8)	8 (12.1)	0.046
Nitrate	8 (33.3)	1 (2.1)	1 (2.0)	16 (24.2)	< 0.001

The values denote mean (standard deviation) or number (percentage)*

CABG: coronary artery bypass grafting; AVR: aortic valve replacement; MVS: mitral valve surgery; PCI-CA: percutaneous coronary intervention or coronary angiography; BMI: body mass index; HDL: high density lipoprotein; LDL: low density lipoprotein; LVEF: Left ventricular ejection fraction; ACE: angiotensin-converting enzyme

Kruskall–Wallis test was used to analyze group differences for numerical variables and Fisher’s Exact test for categorical variables

**Table 2:** Variation in accelerometer measures of physical activity and sedentary behaviour per day in the patient groups

		CABG	AVR	MVS	PCI-CA
Steps (number)	Mean min max SD	4665 1595 9836 2679	6332 812 12,638 3280	6099 1761 13,594 2909	6094 1444 13,322 2597
MVPA (min)	Mean min max SD	22 1.5 51 19	41* 0.9 165 35	35 2.2 112 26	37 1.9 95 23
LPA (min)	Mean min max SD	181 90 387 84	198 71 396 81	213 75 556 86	205 62 411 84
Standing (min)	Mean min max SD	82 26 153 41	72 8.3 178 36	76 18 193 45	98 28 313 56
SB(min)	Mean min max SD	636 472 903 112	605 310 866 129	581 370 767 85	566* 355 767 101
3-minute mean MET level	Mean min max SD	3.5 2.6 4.5 0.7	4.1* 2.7 7.7 1.1	4.1* 2.4 7.9 1.1	4.0* 2.9 6.5 0.6
3-minute peak MET level	Mean min max SD	3.9 2.8 5.2 0.8	4.8* 3.0 10.9 1.5	4.9* 3.3 8.2 1.3	4.7* 3.5 8.5 0.8

The values denote mean, minimum, maximum and standard deviation (SD)

CABG: coronary artery bypass grafting; AVR: aortic valve replacement; MVS: mitral valve surgery; PCI-CA: percutaneous coronary intervention or coronary angiography; MVPA: moderate-to-vigorous physical activity; LPA: light physical activity; SB: sedentary behaviour; MET: metabolic equivalent (3.5 mL/kg/min of oxygen consumption)

*Indicates statistically significant (*p* < 0.05) difference between the patient groups, CABG group as the reference group. The group differences were analyzed with Analysis of Covariance using Sidak-adjustment to correct for multiple comparisons

## Daily PA, standing time and SB in cardiac patients and FinFit2017 participants

**Fig. 3:**
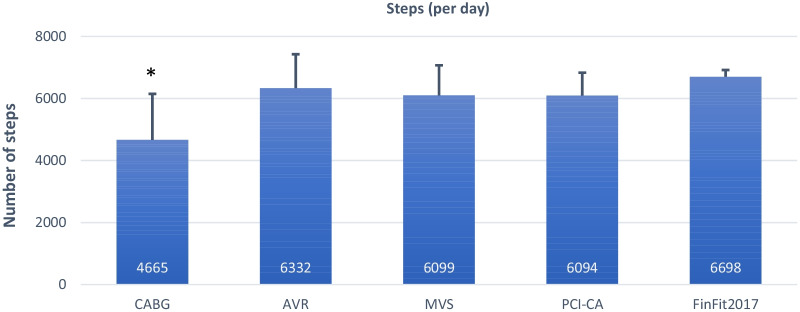
Mean number of daily steps. The values denote mean and 95% confidence interval. CABG: coronary artery bypass grafting; AVR: aortic valve replacement; MVS: mitral valve surgery; PCI-CA: percutaneous coronary intervention or coronary angiography; FinFit2017: population-based sample of 60-69-year-old Finnish adults. *Indicates statistically significant difference (*p* < 0.05) between patient groups and FinFit2017 group (Independent samples t-test assuming that variances are not equal)

When analyzing cardiac patient groups together, the cardiac patients had on average fewer steps per day than the FinFit population (*p* = 0.01). Especially, the CABG group had fewer steps than the FinFit population (*p* = 0.01) (Fig. 3).

When combining all cardiac patients and comparing them to the FinFit population sample, they tended to have on average less accumulated time from MVPA (*p* = 0.06, NS). Especially, the CABG group had significantly less MVPA than the FinFit population (*p* = 0.002). When comparing the cardiac patients, the AVR patients had 86% greater mean time accumulated from MVPA than the CABG patients (*p* = 0.02). There were no statistically significant differences in respect of LPA between the FinFit group and the cardiac patient groups either combined or separately (Fig. 2).

The cardiac patient groups together had significantly less standing than the FinFit population (*p* < 0.001). The AVR group (*p* < 0.001) and the MVS group (*p* < 0.001) spent on average less time standing than the FinFit population. The PCI-CA group stood on average more than the AVR (*p* = 0.01) and MVS groups (*p* = 0.03) (Fig. 2).

The patient groups did not differ statistically significantly from the FinFit population in daily SB. However, when comparing the different patient groups, the CABG group had on average 70 min more daily SB than the PCI-CA group (*p* = 0.03) (Fig. 2).

### Daily accumulation of MVPA, total PA and SB from different bout lengths

**Fig. 4:**
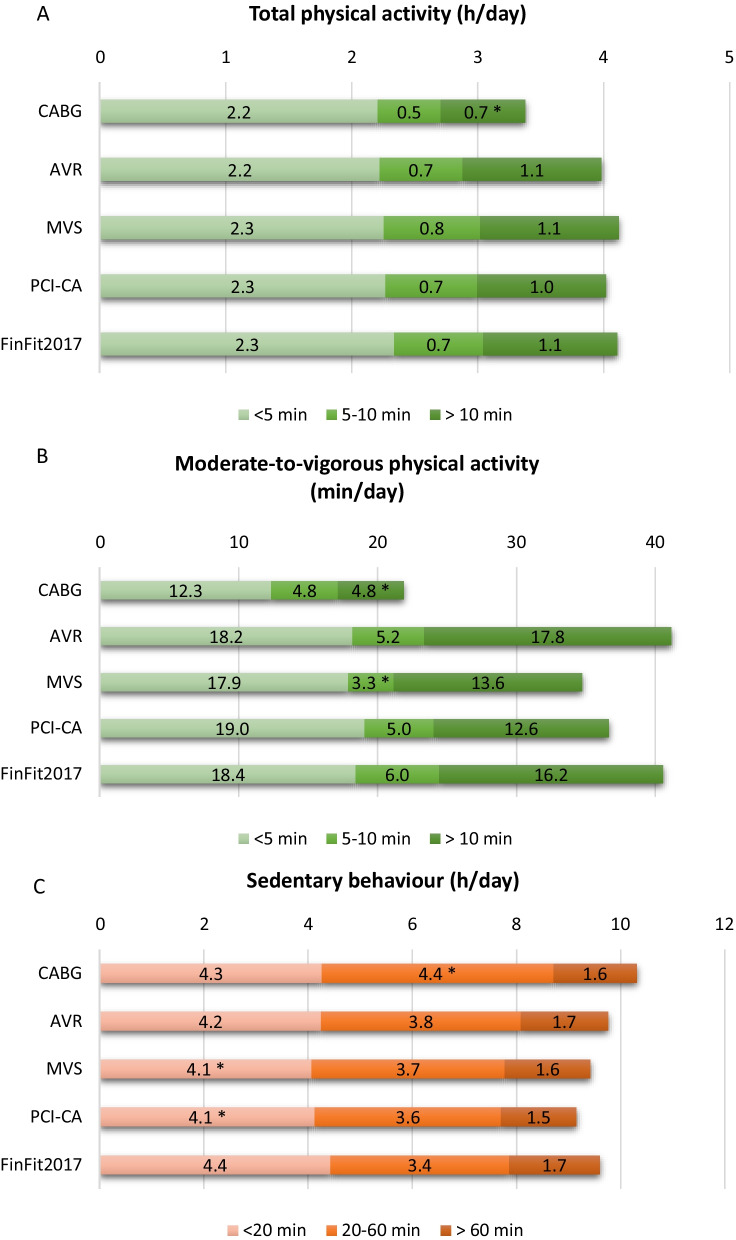
Total physical activity (**A**), moderate-to-vigorous physical activity (**B**) and sedentary behaviour (**C**) accumulating from different bout lengths (mean hours or minutes per day). CABG: coronary artery bypass grafting; AVR: aortic valve replacement; MVS: mitral valve surgery; PCI-CA: percutaneous coronary intervention or coronary angiography; FinFit2017: population-based sample of 60-69-year-old Finnish adults. *Indicates statistically significant difference (*p* < 0.05) between the patient groups and the FinFit2017 group (Independent samples t-test assuming that variances are not equal).

There were no statistically significant differences in total PA bouts between the FinFit population and the combination of all patient groups. However, the CABG group had less total PA from > 10 min bouts than the FinFit group (*p* = 0.03) (Fig. 4).

The four groups combined, cardiac procedure patients had less MVPA accumulating from bouts lasting 5–10 min per day than the FinFit population (*p* = 0.007). This difference was also seen when comparing the MVS group to the FinFit group (p < 0.001). In MVPA accumulating from bouts lasting > 10 min, the CABG group had 71% less MVPA than the FinFit group (p < 0.001). Additionally, the CABG group had less MVPA accumulating from these bouts than the AVR group (*p* = 0.02) (Fig. 4).

The patient groups together had less SB accumulating from < 20 min bouts (*p* = 0.002) and more SB from 20 to 60 min bouts (*p* = 0.002) than the FinFit control group. Especially, the MVS (*p* = 0.04) and PCI-CA (*p* = 0.02) groups accumulated less SB from < 20 min bouts than the FinFit population. The CABG group accumulated more SB from 20 to 60 min bouts than the FinFit group (*p* = 0.02). When comparing the four patient groups, the CABG group had more SB from 20 to 60 min bouts than the MVS (*p* = 0.04) and PCI-CA (*p*= 0.01) groups (Fig. 4).

## Discussion

To the best of our knowledge, this is the first study to investigate various parameters of preoperative PA, standing and SB among patients scheduled for CABG, AVR, MVS or PCI-CA, applying the cutting edge, accelerometer-derived 24/7 measurement technology. We found that the cardiac patients had fewer steps per day than the population-based sample of 60-69-year-old Finnish adults (6004 vs. 6698, respectively). The result is in line with a previous study assessing similar accelerometer-derived parameters, reporting that CVD patients had on average fewer steps per day than their healthy peers [26]. We also found that the CABG group had substantially less total daily MVPA and MVPA accumulating from > 10 min bouts than the FinFit population. This difference was also seen in > 10 min total PA bouts. Regarding SB, the cardiac patients had on average longer bouts than the FinFit population, accumulating more time from 20 to 60 min bouts and less from < 20 min bouts per day.

Of the four patient groups, the CABG patients had the worst activity profile. They had least steps, MVPA and LPA minutes, and the greatest accumulated sedentary time. Additionally, they had significantly fewer steps and less MVPA minutes than the population-based sample. The AVR, MVS and PCI-CA groups had quite similar activity levels. However, the AVR and MVS groups had significantly less standing than the FinFit population and the PCI-CA group.

There are several possible explanations for the present results. The AVR patients had a surprisingly good activity profile despite their severe illness. This may result from AVR patients being regularly monitored for the right moment for anticipated surgery, which is usually scheduled when the first symptoms or signs of impaired left ventricular function arise. Therefore, they have relatively good preoperative functional capability. MVS patients also have the surgery scheduled soon after symptoms or signs of left ventricular dysfunction appear. This may explain their higher activity levels. This study suggests that CABG patients have a high burden of co-morbidities (diabetes, hypertension, obesity etc.) and therefore would benefit lifestyle counselling the most. PCI-CA patients are often thought to be more active than CABG patients due to less diffuse disease (e.g. one or two vessel disease), and these results would suggest so. However, there is a lack of studies investigating the difference in PA between PCI-CA and CABG patients. It is also possible that certain medications (e.g. beta blockers) might affect this comparison between PCI-CA and CABG patients. However, the influence is propably small, as the percentages of patients using these drugs in both groups are quite similar.

Less than five minutes was the bout length with the longest accumulated mean time in total PA and MVPA, which indicates that short periods of activity are of great importance in cardiac procedure patients and a possible target for interventions. The importance of shorter bouts of PA has been recognized only recently [27, 28], while the former recommendations acknowledged PA lasting longer than 10 min [29]. In addition, it is very difficult, if not impossible, to remember, report and calculate all short bouts of PA when assessing one’s own PA, which highlights the importance of objective measurements.

While the bout length with the longest accumulated mean time of SB was < 20 min, interestingly, 20–60 min was the length with the greatest variation between the different groups. Reducing and breaking these SB bouts, as well as replacing them with PA, could have positive impact on health and potentially postoperative outcomes [9, 30]. The SB of FinFit population consisted of shorter bouts than that of the cardiac patients. It should be investigated whether the incoming operation increases the sedentary time of cardiac patients.

The individual variance in daily activity levels within the patient groups was large. There were patients with very low activity levels. On the other hand, some patients were surprisingly active before their operation. This was seen for example in daily steps and MVPA, e.g. the eighteenfold difference in the AVR group between longest and shortest accumulated MVPA minutes. Additionally, large differences in the mean and maximum daily MET levels indicate varying energy consumption. Thus, based on the current results, the postoperative rehabilitation program should be tailored individually.

We chose the objective approach to measure PA and SB, because self-reported and objective assessment of PA and SB are not comparable, and there is a lack of knowledge about objectively measured preoperative PA and SB [31, 32]. In addition, analyzing the data with MAD and APE algorithms provides information about PA and SB that is precise and can be used regardless of the accelerometer brand [21–23]. The patients who had both valve surgery and CABG, were included in the valve group. This was also done in a study by Noyez et al. 2013 [19]. The differences between patient groups did not significantly change whether the combined operation patients were included in the valve surgery groups or not.

There are several strengths in this study. The patient samples from the four procedures were inclusive and we used the wide reference group of 60-69-year-old population sample from the FinFit2017 study that has identical data collection and analyses. The parameters used for PA and SB assessment are universal and accurate, and 24/7 measurements were comprehensive. However, certain limitations are to be acknowledged also. A minor loss of PA from water activities was due to the fact that the accelerometer was advised not to be exposed to water. Further, any causative influence cannot be recognized, as the study design is cross-sectional. Although the number of patients was somewhat greater than in most of the studies investigating interventions in cardiac rehabilitation published so far [30, 33], the interpretation of these results should be done cautiously.

Because CVDs are a major economic burden to the health care, exercise-based interventions provide a cost-effective way of alleviating it [34]. Also, preoperative interventions for cardiac patients, with for example an accelerometer or applications, might reduce postoperative complications [35–37]. Therefore, both pre- and postoperative rehabilitation to increase physical activity and reduce sedentary behaviour could improve postoperative recovery.

## Conclusions

Patients scheduled for elective cardiac procedure had fewer daily steps than the FinFit population sample. Of the different procedure types, the CABG group had least MVPA and most SB, and had less total MVPA and MVPA accumulating from > 10 min bouts than the FinFit population. In addition, there was large variation among all patient groups in terms of preoperative PA and SB, which might potentially influence the recovery after the operation and could be used to individualise the rehabilitation program. By measuring various parameters of PA and SB with high accuracy, we uncovered possible aspects of the activity profiles of patients scheduled for cardiac procedures that could be targeted in future interventions both pre- and postoperatively.

## Data Availability

The datasets generated during and analyzed during the current study are not publicly available due to the privacy of research participants but are available from the corresponding author on reasonable request.

## Abbreviations

PA: Physical activity
SB: Sedentary behaviour
PCI-CA: Percutaneous coronary intervention or coronary angiography
CABG: Coronary artery bypass grafting
AVR: Aortic valve replacement
MVS: Mitral valve surgery
MET: Metabolic equivalent
LPA: Light physical activity
MVPA: Moderate-to-vigorous physical activity
CVD: Cardiovascular disease
MVR: Mitral valve replacement
MVP: Mitral valve repair
MAD: Mean amplitude deviation
APE: Angle for posture estimation
PACO: Personalized intervention to increase physical activity and reduce sedentary behaviour in rehabilitation after cardiac operations

## Declarations

The Research Ethics Committee of the Northern Savo Hospital District provided the ethical approval for this study (304/2017). All participants signed a written informed consent before participation. The trial is registered in clinicaltrials.gov as NCT03470246 (19/03/2018).

An ethical approval for the FinFit2017 study was provided by The Regional Ethics Committee of the Expert Responsibility Area of Tampere University Hospital (R17030) and the participants signed a written informed consent before participation.
